# A data-driven crop model for maize yield prediction

**DOI:** 10.1038/s42003-023-04833-y

**Published:** 2023-04-21

**Authors:** Yanbin Chang, Jeremy Latham, Mark Licht, Lizhi Wang

**Affiliations:** 1 Department of Industrial and Manufacturing Systems Engineering, Iowa State University, 2529 Union Drive, Ames, 50011 IA USA; 2 Department of Agronomy, Iowa State University, 716 Farm House Lane, Ames, 50011 IA USA

**Keywords:** Plant sciences, Physiology

## Abstract

Accurate estimation of crop yield predictions is of great importance for food security under the impact of climate change. We propose a data-driven crop model that combines the knowledge advantage of process-based modeling and the computational advantage of data-driven modeling. The proposed model tracks the daily biomass accumulation process during the maize growing season and uses daily produced biomass to estimate the final grain yield. Computational studies using crop yield, field location, genotype and corresponding environmental data were conducted in the US Corn Belt region from 1981 to 2020. The results suggest that the proposed model can achieve an accurate prediction performance with a 7.16% relative root-mean-square-error of average yield in 2020 and provide scientifically explainable results. The model also demonstrates its ability to detect and separate interactions between genotypic parameters and environmental variables. Additionally, this study demonstrates the potential value of the proposed model in helping farmers achieve higher yields by optimizing seed selection.

## Abstract (web summary)

A data-driven and process-based model incorporates machine learning to predict maize yield from available historical data over both temporal and spatial dimensions using explainable parameters without the need for experimental calibration.

## Introduction

Predicting crop yield is central to addressing emerging challenges in food security, particularly in an era of global climate change^1,2^. Accurate yield predictions help farmers make informed economic and management decisions and can support famine-prevention efforts and the global food security. Early crop model pioneers have developed research^3–5^ to categorize many relevant factors that are needed by crop models, such as temperature, humidity and leaf area index (LAI). To date, underlying yield prediction is one of the greatest challenges of biology: understanding how phenotype is determined by genotype, environment, and their interactions. Specifically, the relationship between genetics, weather, soil and management variables and crop yield has been the subject of extensive studies^6–16^. Pursuing more accurate crop yield prediction techniques has and will continue to motivate innovation at the intersection of plant science and data analytics.

Majority of the literature on crop yield prediction falls into two categories: processed-based crop models and data-driven machine learning models, both of which have their salient strengths and weaknesses. Process-based crop models, such as APSIM^17–19^ and DSSAT^20–22^, describe the crop growth process and development as a complex function of weather, soil, and management. As such, process-based crop models reflect human knowledge of plant biology and are easily explainable in terms of physiological mechanisms. For example, Yield Prophet^23^, an APSIM-based online crop simulation service, was set up to help farmers avoid over- or under-investing in their crops by forecasting potential yields with detailed inputs such as nitrogen application types and altered sowing dates. Since crop models can be experimentally validated, their results provide not only crop yield predictions but also scientific explanation of such predictions. However, these models face several serious challenges. Calibration of the numerous parameters of the a crop model typically requires time-consuming and resource intensive field experiments, yet these parameters are hardly generalizable across different varieties and environmental conditions. Oftentimes, the large variability of environmental conditions, coupled with choices of model structure and parameters, limits the predictive performance of these models beyond the spatiotemporal variability of observed yields in a large area^24,25^. Application of process-based crop models is also limited by the paucity of spatially detailed input data^24^. In the absence of spatial data on the distribution of key model inputs such as information on crop cultivars and management (e.g. irrigation, planting, fertilization, tillage, weed control), modelers often make broad assumptions across large geographic regions that may or may not reflect on-the-ground decision-making of individual producers in the context of economic opportunities and policy incentives^26^.

In contrast to the process-based methodology, machine learning models take a data-driven approach to approximate the complex relationship between input (genotype and environment) and output (crop yield) without relying on human knowledge on crop science, which is incomplete and sometimes incorrect. Several machine learning models have been successfully deployed to produce remarkable prediction accuracy, including multiple linear regression^27^, partial least squares regression^28^, random forest regression^29,30^, convolutional neural networks^31^, deep neural network^10,32,33^, among others. The sophisticated and powerful model structures of these data-driven models, when trained with high quality large datasets, are able to implicitly account for both additive effects and interactions among genotype, environment, and crop management practices, allowing them to outperform most crop models in terms of prediction accuracy. Some satellite-based indicators have also been utilized in the data-driven crop model to study the crop yield in a large area, such as Gross Primary Productivity (GPP)^34^, Normalized Difference Vegetation Index (NDVI)^35–37^, and Enhanced Vegetation Index (EVI)^38,39^. Some recent research has incorporated the remotely sensed data derived indicators into the machine learning crop model^40–45^. However, these models also inevitably suffer from the common limitations of machine learning models. They are sensitivity to data quantity and quality^46^, limiting their applicability to crops with sufficient datasets. Machine learning models often include a huge number of parameters in a blackbox structure, but it is hard to discern how the parameters are used to incorporate input data into the model to predict a particular outcome such as crop yield; as such, it is difficult to extract scientific insights from the results or transfer them spatially, temporally, or genetically^47–49^.

An emerging and promising research direction is to integrate process-based models and data-driven ones. Huang et al.^50^ used Bayesian averaging method to construct a process-based ensemble model to provide a reliable maize yield forecast in Liaoning Province, China. Feng et al.^51^ combined the APSIM and statistical regression-based model to improve the accuracy of wheat yield prediction by dynamically tracking climate and remote sensing indices during the growing season. Shahhosseini et al.^52^ integrated the APSIM model and machine learning models and achieved improved yield prediction accuracy. Saha et al.^53^ used regression-based machine learning models integrated with the crop growth model to improve the prediction of temporal nitrous oxide emissions from corn and soybean in the Midwest of the United States.

**Fig. 1:**
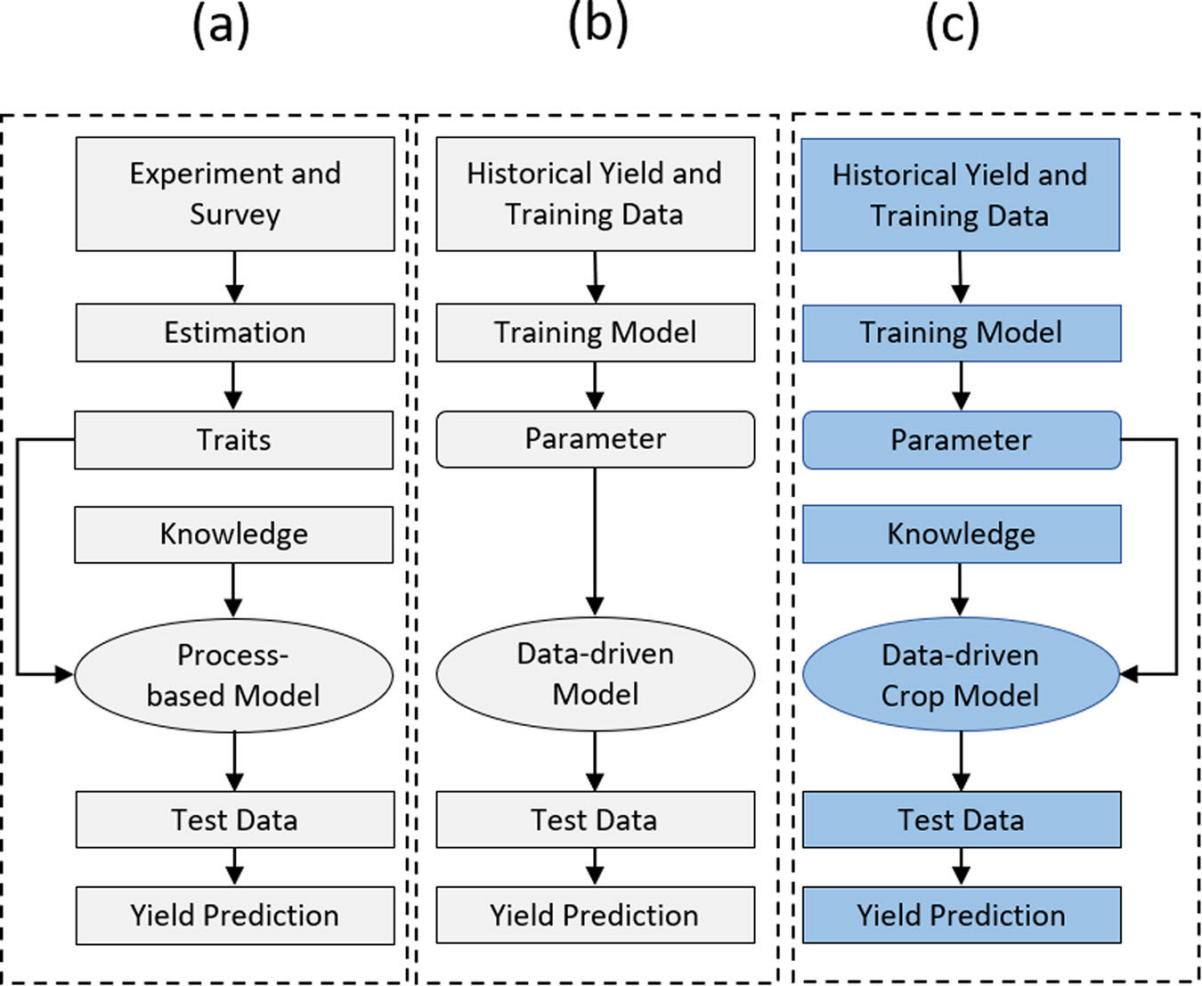
Comparison of process-based, data-driven, and the proposed data-driven crop models. **a** Process-based models are built with human knowledge on plant physiology with explicit assumptions about genotype by environment interactions; numerous traits (modeling parameters) need to be estimated using experiments or survey of the literature. **b** Data-driven models rely on historical data to approximate the complex relationship between input and output. **c** The proposed Data-driven Crop model combines the strengths of two types of models.

In this paper, we present a data-driven crop model for maize in an attempt to combine the strengths of process-based models with those of data-driven models and overcome their limitations. The proposed model attempts to provide explanatory crop yield predictions with the available historical data over both temporal and spatial dimensions without the need for experimental calibration. The proposed model uses a crop model to describe how crop yield is determined by genotype, environment, and their interactions; data-driven techniques are used to calibrate model parameters from historical data. Figure 1 illustrates how the data-driven crop model (subfigure c) conceptually differs from a process-based model (subfigure a) and a data-driven model (subfigure b). Similar to the process-based model, the data-driven crop model also describes plant phenotype as a result of genotype, environment and their interactions throughout the crop growth process, preserving the advantage of being scientifically explainable and insightful. There are three major differences between the proposed data-driven crop model and other existing crop models in the literature. First, the data-driven crop model defines the genetic properties as parameters for each crop variety. In contrast, some parameters used in conventional crop models (e.g., LAR and LAI in APSIM) are jointly determined by genotype and environment. Being independent from environmental effects, the genotypic parameters in the data-driven crop model are transferable to other environments, whereas the parameters for other crop models may need to be re-calibrated when the same varieties are grown in a different environment. Second, the data-driven crop model is designed to be a flexible framework that consists of a number of modules to reflect the crop growth process. The composition of these modules depends on the availability of data. Conventional crop models have a fixed requirement of datasets; as a result, missing or unavailable data must be imputed or assumed before the modeling can be used^54^. Third, rather than relying on large amount of field experiments for parameter calibration, the data-driven crop model employs machine learning methods to train the parameters to best fit historical data within reasonable ranges.

## Result

In order to demonstrate the effectiveness of the data-driven crop approach, we applied the descriptive and predictive models to the dataset described in next Method section. Computational experiments were conducted using the Python on a laptop with an Intel i7-10750H processor running at 2.60 GHz with 16 GB of RAM.

### Training accuracy

**Fig. 2:**
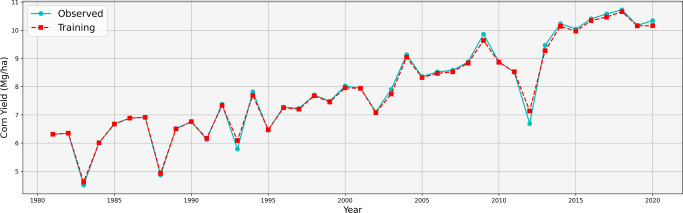
Training performance of proposed model. The cyan and red curves are, respectively, observed yield and fitted yield using training data between 1981 and 2020, averaged across all counties in the Corn Belt.

We were able to calibrate the genotypic parameters to achieve an RMSE of 0.74 Mg/ha for the training data; with respect to the average yield in 2020 in the Corn Belt (10.34 Mg/ha), the relative RMSE (or RRMSE) was 7.16%. Figure 2 shows the observed and fitted yields between 1981 and 2020. The overall training accuracy in the last decade was slightly higher than the first three; low accuracy years were often accompanied by extreme weather, such as the great flood in 1993 and the drought in 2012.

To benchmark the modeling performance, we found two deep learning models published in 2019^10^ and 2020^11^ using similar Corn Belt datasets. Their training RMSEs were 0.67 Mg/ha and 0.72 Mg/ha, respectively. In a more recent study^52^, a new model was proposed that combined machine learning and APSIM models, and their training RMSE was 0.69 Mg/ha using a similar dataset. Therefore, the data-driven crop model demonstrated its capability to reach a comparable prediction accuracy with state-of-the-art models in the literature.

### Spatial extrapolation

**Fig. 3:**
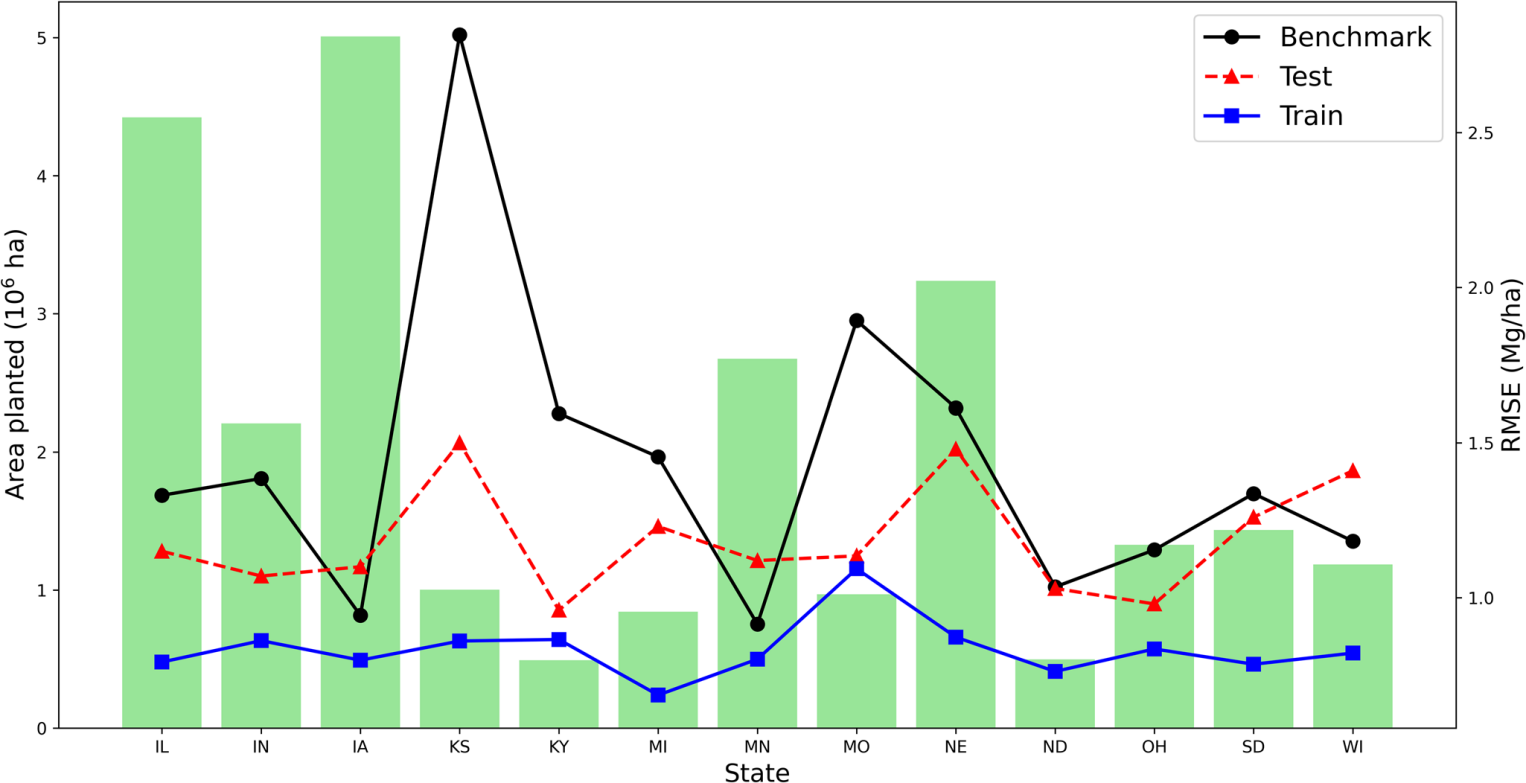
Spatial extrapolation results. Green bars are average planted areas from 1981 to 2020. The three curves represent the benchmark nearest-county approach on the test data, and the data-driven crop model on test data and training data.

To evaluate the predictive performance of a trained data-driven crop model on an unseen location, we conducted thirteen experiments. In each experiment, we first select a county *c* in the test state, carving out all data of county *c* from the training data and using them as test data. After obtaining the predictive performance of the previously unseen county *c*, we move to the next county in the test state until the process is complete for all counties in the test state. The nearest-county approach was used as a benchmark prediction strategy: the historical yield for the nearest county to county *c* in year *t* was used as the predicted yield for the unseen county *c* in year *t*; the planted area weighted average predicted yield for all counties from 1981 to 2020 in the test state was then used to compare with the observed average yield in the test state. Figure 3 plots the RMSEs of the benchmark approach, the data-driven crop prediction on the test data and training data, as well as the planted areas.

The average RMSE and RRMSE for spatial extrapolation were 1.17 Mg/ha and 11.32%, respectively. In contrast, the benchmark RMSE and RRMSE were 1.44 Mg/ha and 13.93%, respectively; the training RMSE and RRMSE were 0.83 Mg/ha and 8.03%, respectively. Nebraska and Kansas had the highest RMSEs, which may be partly due to a lack of irrigation data. The descriptive modeling assumes zero irrigation, given that many states in the Corn Belt are rainfed and no irrigation data are available. However, Nebraska and Kansas were among the most irrigated states in the Corn Belt, which could lead to higher prediction errors. These results suggest that the predictive performance of the model could be further improved with additional irrigation data.

### Temporal extrapolation

**Fig. 4:**
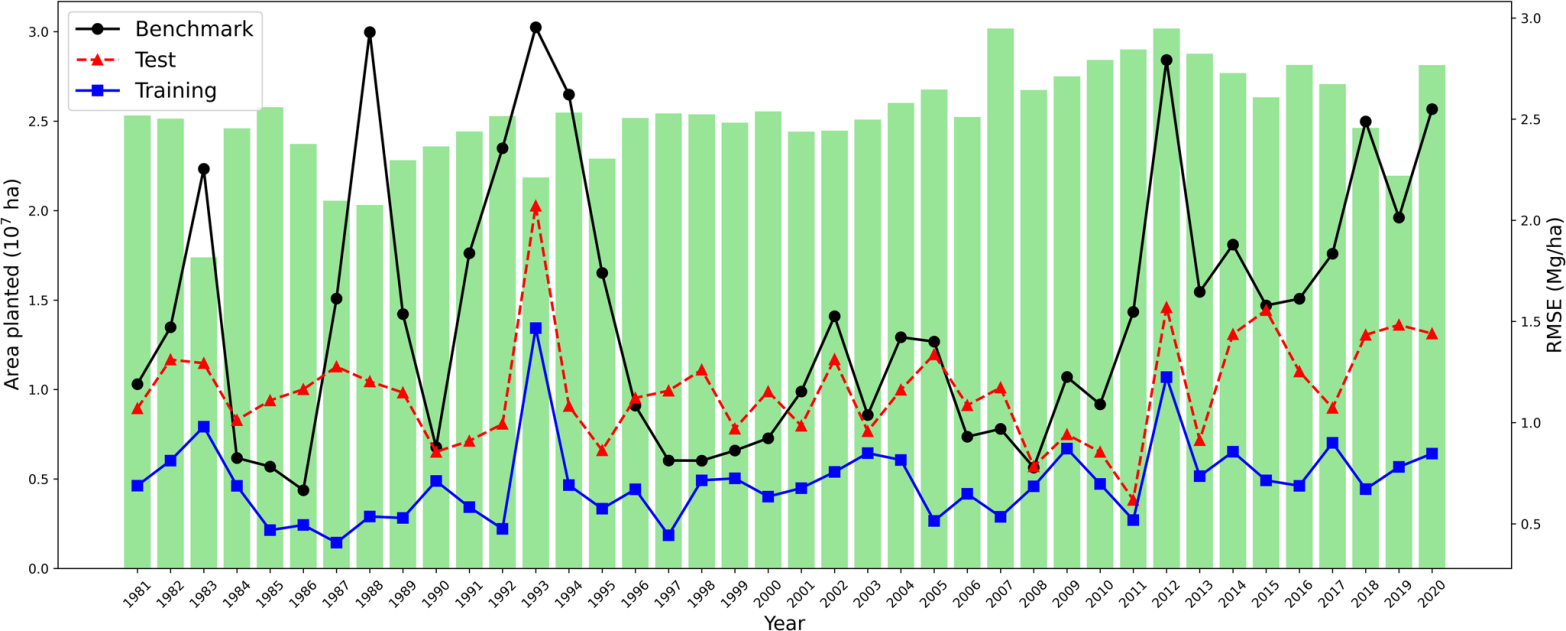
Temporal extrapolation results. Green bars are average planted areas from 1981 to 2020. The three curves represent the benchmark nearest-year approach, data-driven crop model on test data and training data.

Similar to spatial extrapolation, we also evaluated the temporal extrapolation of the data-driven crop model. We carried out forty experiments, each time carving out all data for one year between 1981 and 2020 from the training data and using them as test data. The nearest-year approach was used as a benchmark prediction strategy: the average historical yield for county *c* in years *t* − 1 and *t* + 1 was used as the predicted yield for county *c* in the unseen year *t*; the planted area weighted average predicted yield for all counties in the test year was then used to compare with the observed average yield in the test year. Figure 4 plots the RMSEs of the benchmark approach, the data-driven crop model prediction on the test data and training data, as well as the planted areas.

The average RMSE and RRMSE for temporal extrapolation were 1.15 Mg/ha and 11.12%, respectively. In contrast, the benchmark RMSE and RRMSE were 1.55 Mg/ha and 14.99%, respectively; the training RMSE and RRMSE were 0.71 Mg/ha and 6.87%, respectively. The benchmark approach struggled in drought (1983, 1988, 2012) or flood (1993) years. The data-driven crop model performance improved during the aforementioned years, although 1993 was still more challenging than other years. These results suggest the direction of improving predictive performance by refining the design of the stress module in the descriptive model.

### Genotype by environment interactions

Since the genotypic parameters in the data-driven crop model were defined to be solely determined by the genotype and independent of environmental effects, the model is able to answer “what-if” questions regarding genotype by environment interactions.

In this experiment we explore the hypothetical scenarios of growing all the historical seeds under all historical weather conditions. To estimate yields in all of these scenarios, we extracted genotypic parameters for all states and all years and combined them with environmental and management data to produce the predicted yield for the desired combination. For example, the predicted yield of growing genotype from year *t*_1_ in the environmental conditions of year *t*_2_ in county *c* is calculated from function $f({W}_{{t}_{2},c},{M}_{{t}_{2},c},{S}_{c},{g}_{{t}_{1},c},{s}_{c})$.

**Fig. 5:**
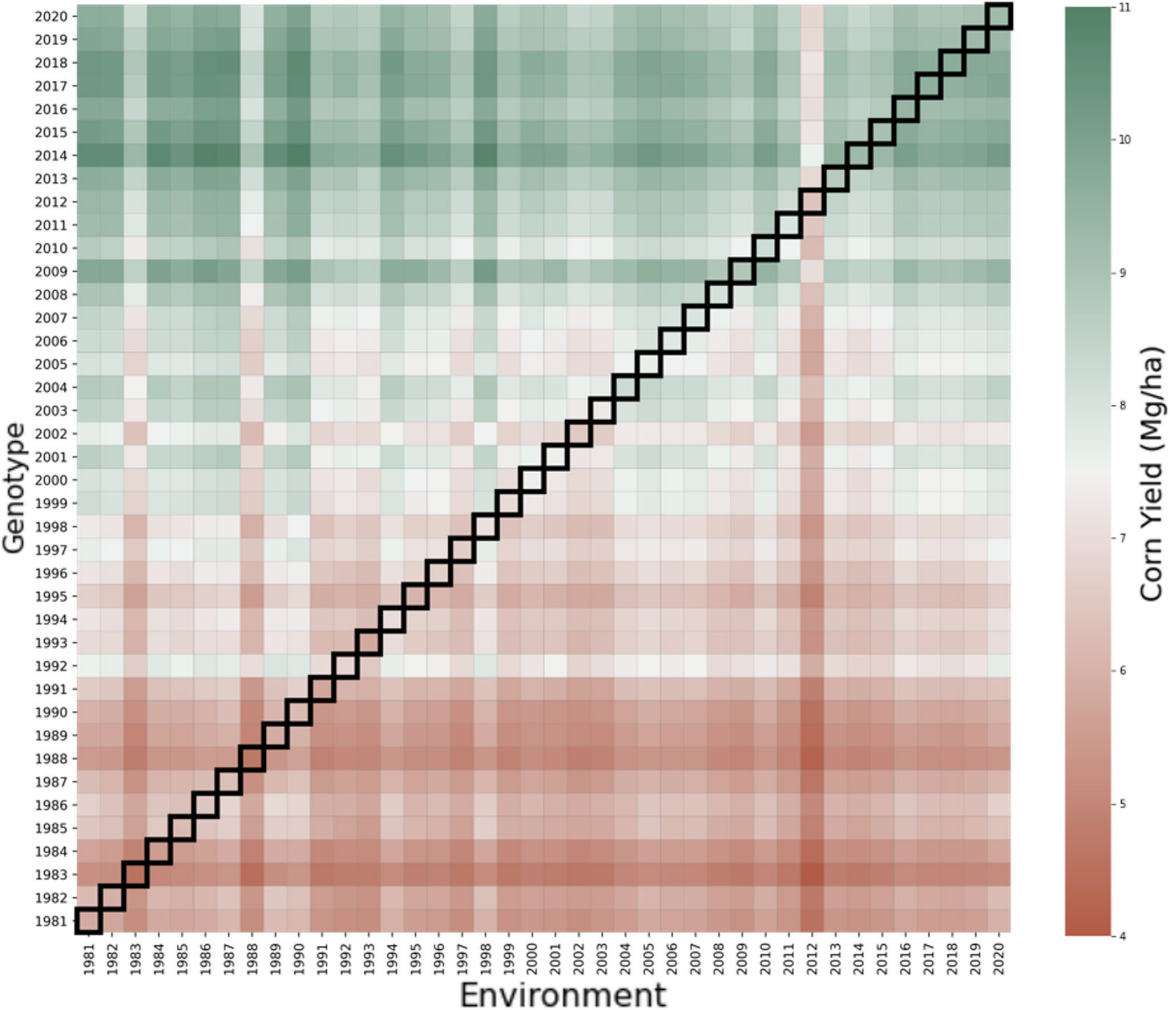
Genotype by environment interactions result. Each colored square in the heat map indicates the predicted yield using the data-driven crop model when the genotype in the year from its corresponding vertical axis was grown in the environmental conditions in the year from its corresponding horizontal axis.

Results for this analysis were presented in Fig. 5, where the horizontal axis is the environmental conditions (weather, soil, management) from 1981 to 2020 averaged across all counties in the Corn Belt, and the vertical axis is the genotypic parameters from 1981 to 2020 averaged across all Corn Belt counties. Each colored square indicates the predicted yield of growing a given genotype under a given set of environmental conditions averaged over all counties in the Corn Belt. Diagonal thick squares represented the actual observed historical scenarios with genotype and environments belonging to the same years, while the other colored squares represented predicted yields of other hypothetical combinations. The lower triangle answers the question of “what if historically available seeds were grown in subsequent years?”, which could potentially have been carried out given sufficient resources; whereas the upper triangle answers the question of “what if future seeds were brought back and grown in historical years?”, which would not be physically possible without a time machine. The answers to both types of what-if questions provide insights into the evolution of seed genotype, environmental conditions, and their interactions over the past four decades. For instance, the 2012 drought was so devastating that no seeds in the past four decades could have produced much better; whereas the genotype of the seeds since 2009 have improved so much that they would have resulted in much higher yields if the same environmental conditions from 1981 to 2018 were to be repeated.

### Yield improvement from optimal seed selection

In this experiment, we demonstrate the potential yield improvement from optimal seed selection. For the growing season in year *t* in county *c*, suppose all seeds for all counties in the Corn Belt from 1981 to *t* are available, then results from training can be used to select the optimal seed to maximize the yield. Here we consider two scenarios, one that assumes a complete knowledge of the weather in year *t* at the time of seed selection, representing a more optimistic scenario, and another that assumes zero additional knowledge of weather in year *t* beyond historical weather data, which is a more realistic scenario.

The seed selection problem for county *c* in year *t* under known weather can be formulated as the following optimization model:

1 $$\mathop{\max }\limits_{d,r}f({W}_{t,c},{M}_{t,c},{S}_{c},{g}_{r,d},{s}_{c})$$

2 $$d\in {{{{{{{\mathcal{C}}}}}}}}$$

3 $$r\in \{1981,1982,...,t\},$$

where ${{{{{{{\mathcal{C}}}}}}}}$ is the set of all counties in the Corn Belt. The objective function (1) is to maximize the predicted yield in county *c* in year *t* by selecting the optimal genotype from county *d* in a historical year *r*, which was simulated using Eq. (7) with detailed definitions in Supplementary Note 3. Constraints (2) and (3) set the limits on county *d* and historical year *r*, respectively.

The seed selection problem for county *c* in year *t* under unknown weather can be formulated as the following optimization model:

4 $$\mathop{\max }\limits_{d,r}\frac{1}{t-1981}\mathop{\sum }\limits_{\tau =1981}^{t-1}f({W}_{\tau ,c},{M}_{\tau ,c},{S}_{c},{g}_{r,d},{s}_{c})$$

5 $$d\in {{{{{{{\mathcal{C}}}}}}}}$$

6 $$r\in \{1982,1983,...,t\},$$

Here, the objective function (4) is to maximize the expected predicted yield in county *c* in year *t* under all historical weather conditions by selecting the optimal genotype from county *d* in a historical year *r*, with the ranges of *d* and *r* being specific by Constraints (5) and (6), respectively.

**Fig. 6:**
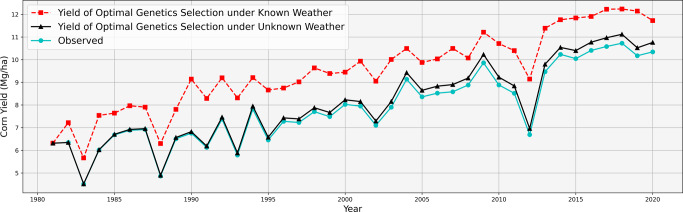
Comparison of observed corn yield with improved yield from optimal seed selection under known and unknown weather scenarios. In both scenarios, optimal genotype were selected from all seeds in all counties in the Corn Belt that were historically available at the time of selection.

Results are presented in Fig. 6, with the observed annual yield (averaged across all counties in the Corn Belt), improved yield with known weather using model (1) and (2), and improved yield with unknown weather using model (3) and (4) are all plotted in the same figure. The overall yield benefit trend is increasing over time due to the increased pool of historically available genotype since 1981. The average observed yield from 2011 to 2020 across all counties in the Corn Belt was 9.72 Mg/ha, whereas optimal seed selection would have been able to achieve an additional 0.38 Mg/ha, which was 3.91% of the average observed yield. With perfect meteorological insight, such yield improvement would have become 1.73 Mg/ha and 17.59%.

Results from this experiment demonstrated the potential value of the data-driven crop model for prescriptive analysis, which would not have been possible without its descriptive ability to separate the genotypic and environmental effects of crop yield and its predictive capability to answer what-if questions.

## Discussion

In an attempt to combine the complementary strengths of process-based models and data-driven models and overcome their limitations, we proposed a data-driven crop model for maize yield prediction; this model has several salient features. First, its descriptive modeling framework adopts a crop model structure without the need for experimental calibration. As such, the modeling results are scientifically insightful and explainable. Second, its predictive modeling framework is able to extract knowledge from historical data without using a blackbox modeling structure. Since all modeling parameters are biologically meaningful, the training process is less sensitive to the quantity and quality of the training dataset. Third, the model is capable of providing prescriptive insight due to the clear separation of genotypic parameters from environmental variables and explicit descriptions of their interactions.

A comprehensive county-level dataset for the Corn Belt was used to demonstrate the performance of the data-driven crop model in our computational experiments. Many factors (such as waterlogging) are assumed to be uniform across the county. Results showed that the model was able to fit the historical data with a 7.16% RRMSE, and its spatial and temporal extrapolation RRMSEs are 11.32% and 11.12%, respectively. These predictive performances are competitive against the state-of-the-art crop yield prediction models. The data-driven crop model also predicted the yield of all combinations of historically available genotype and environmental conditions using insights from genotype by environment interactions. Additionally, the model demonstrated its prescriptive value in maximizing predicted returns through optimal seed selection. Our results indicated that optimal seed selection would have increased the average yield between 2011 and 2020 by 17.59% and 3.91%, respectively, with and without perfect weather predictions, under the optimistic assumption that all historically available seeds would be available in all counties in all subsequent years.

The proposed model is not without its limitations. For example, prediction errors were particularly large under extreme weather years such as 1983, 1988, 1993, and 2012. The transferability of a modeling structure from one crop species to another is low, since each crop has its unique physiological properties that need to be reflected by a carefully designed new modeling structure. Furthermore, the model relies on some data (such as irrigation and fertilization) that are hard to find or only available at reduced resolution (such as plant population density, planting and harvesting times).

Several future research directions are worth pursuing. First, the data-driven crop model framework needs to be developed and validated for other crop species. Second, more comprehensive case studies should be conducted using a more complete and higher resolution dataset. Third, results from optimal seed selection need to be validated experimentally. Fourth, results from the data-driven crop model, such as the genotypic parameters, may provide useful information for plant breeders.

## Method

In this section, we describe the data-driven crop model for maize yield prediction. The modeling framework, however, may apply to other crop species with an appropriately selected crop model for such species and available data.

### Data

We collected data for the US Corn Belt, which is an important agricultural region, accounting for approximately 87% of the total US corn production and 31% of global production in 2021^55^. Here, we briefly describe the data in different categories. More details are provided in Supplementary Note 1.

#### Yield and geographic data

County-level corn yield in the Corn Belt area from 1981 to 2020 were collected from USDA^56^. After excluding missing values, 47,710 county-year combinations yield data were recorded. Shape files of counties were collected from the National Weather Service^57^. This information was used to determine the membership of counties in crop reporting districts and states. Shape files were also used to locate weather stations and soil map units for calculating average weather and soil variables within each county.

#### Weather data

Daily surface weather data on a 1-km grid from 1981 to 2020 were collected from Daymet^58^.

#### Management data

All management data for counties in the Corn Belt area were collected from USDA^56^. The plant and harvest dates were derived from the data from the state-level crop growth process taking into account the agricultural districts. The corn plant population density (number of plants per acre) data was also at the state level with over 50% of missing value. We utilized the mean of non-missing data (e.g., other years for the same state, if available) for data imputation.

#### Soil data

Soil data were collected from the latest version of the Gridded Soil Survey Geographic (gSSURGO) Database released in July 2020^59^.

### The descriptive modeling framework

Here we present a data-driven crop model for maize, which is tailored to the available weather, soil, and management data. Several major simplifying assumptions were necessary to account for data that were either lacking or only available at low resolution. First, due to unavailable genotype data, we assume that all seeds in each county each year were collectively represented by a unique genotype. As such, these genotypic parameters shed light on temporal and spatial trends in the average genetic performance of commercially available seeds. Second, due to lack of fertilization and irrigation data, we assume that crops were grown without irrigation but under the appropriate fertilizer availability. It is worth noting that the modeling framework does have the ability to incorporate genotype, irrigation, and fertilization data into the crop model should they become available.

**Fig. 7:**
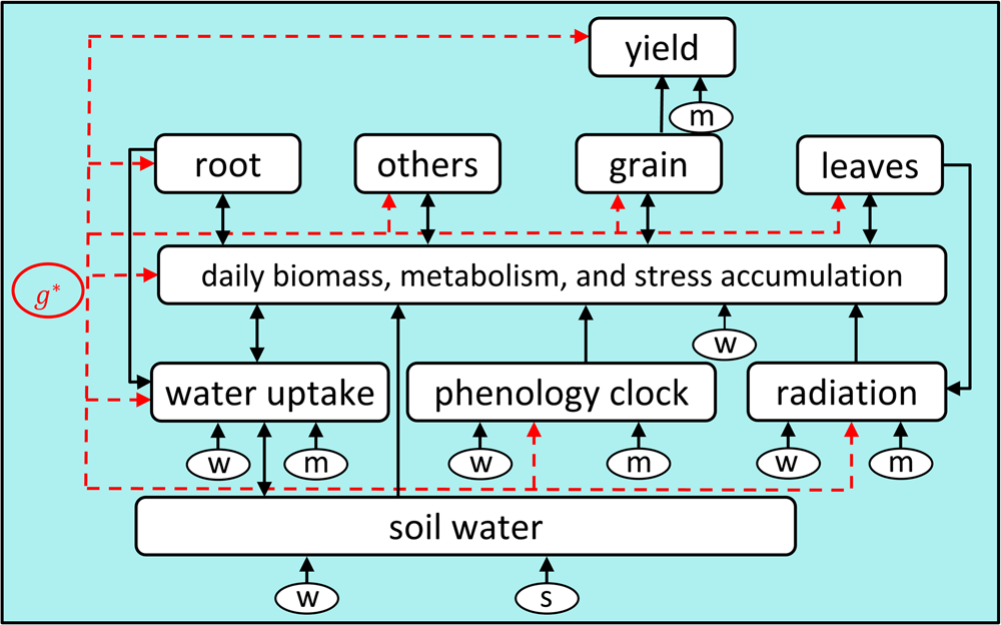
Illustration of a simplified maize growth model. Here “w”, “s”, “m” represent weather, soil, and management variables, respectively, and “g” represents the set of parameters that are determined solely by the genotype and independent of the environment. The arrows indicate how environmental variables and genetic parameters influence different modules and eventually determine crop yield.

Figure 7 illustrates the major modules in the corn crop model, which are briefly described as follows. More details are provided in Supplementary Note 3.

- **Soil water:** Daily soil water levels are affected by precipitation, runoff, crop water uptake, and evaporation.
- **Water uptake:** Daily amount of water uptake is proportional to root mass and atmospheric vapor pressure deficit.
- **Radiation interception:** Daily amount of solar radiation interception is proportional to LAI.
- **Phenology clock:** The growth process of maize can be separated into two growth stages: vegetative and reproductive. The transition occurs when a hybrid specific growing degree daily threshold has been reached.
- **Daily biomass and metabolism:** Daily biomass accumulation is determined by water uptake, solar radiant and leaf weight. Daily metabolism is influenced by crop weight and stress.
- **Stress:** Heat, drought, and flooding stresses are considered. Water deficits caused by heat and drought stresses reduce the amount of soil water available for plant uptake and transpiration, radiation use efficiency, and eventually growth will also be reduced.
- **Crop organs:** In the vegetative stage, certain proportions of daily biomass accumulation are allocated to leaves, roots, and other plant organs; during the reproductive stage, grains begin to fill and leaves and roots cease to grow.

### The predictive modeling framework

We use the following function to represent the descriptive model:

7 $${\hat{y}}_{t,c}=f({W}_{t,c},{M}_{t,c},{S}_{c},{g}_{t,c},{s}_{c}).$$

Here,

- ${\hat{y}}_{t,c}$ is the predicted yield for county *c* in year *t*,
- *W*_*t*,*c*_ is the weather data for county *c* in year *t*,
- *S*_*c*_ is the soil data for county *c*, which is assumed to be static over time,
- *M*_*t*,*c*_ is the management data for county *c* in year *t*,
- *g*_*t*,*c*_ is the genotypic parameter for county *c* in year *t*,
- *s*_*c*_ is the soil parameter for county *c*, and
- *f*(⋅) is the complex function defined in Supplementary Note 3 that describes the complex relationship between input (genotype, weather, soil, management) and output (corn yield), which was hypothesized based on human knowledge in plant physiology and our simplifying assumptions. Detailed variable definitions can be found in Supplementary Note 2.

A key component of the predictive modeling framework is the calibration of *g*_*t*,*c*_. Rather than experimentally estimating such parameters as most traditional crop models do, the data-driven crop model uses historical data to identify the optimal set of genotypic parameters to produce the best fit between predicted yield and observed yield. The calibration of genotypic parameter *g* can be formulated as the following optimization problem. A heuristic algorithm that describes how to solve the data-driven crop model is presented at the end of Supplementary Note 3. Also, definitions of other variables are located in Supplementary Note 2.

8 $$\mathop{\min }\limits_{g,s}\sqrt{\frac{{\sum }_{(t,c)}{\left({M}_{t,c}^{{{{{{{{\rm{area}}}}}}}}}\right)}^{2}{\left({y}_{t,c}-{\hat{y}}_{t,c}\right)}^{2}}{{\sum }_{(t,c)}{\left({M}_{t,c}^{{{{{{{{\rm{area}}}}}}}}}\right)}^{2}}}$$

9 $${\hat{y}}_{t,c}=f({W}_{t,c},{M}_{t,c},{S}_{c},{g}_{t,c},{s}_{c})$$

10 $${g}_{{t}_{1},c}\le 1.25{g}_{{t}_{2},c}\qquad \forall c,{t}_{1},{t}_{2}$$

11 $${g}_{t,{c}_{1}}\le 1.25{g}_{t,{c}_{2}}\qquad \forall {c}_{1},{c}_{2},t.$$

The objective function (8) is to minimize the root-mean square error (RMSE) between predicted and observed yields weighted by planting areas. Equation (9) defines the complex function that produces the predicted yield. Constraints (10) and (11) limit, respectively, the temporal and spatial ranges of the genotypic parameters, which not only help avoid overfitting but also better reflect the fact that changes in genotype are usually gradual. The upper bound ratio of 1.25 between any two counties or years was arbitrary, yet our computational results have shown that the model is insensitive to such ratio.

The optimization model (8)–(11) serves as a data-driven training process, which not only removes the need for experimental calibration of the genotypic parameters (like typical process-based models have) but also enhances the predictive performance of the model, as will be shown in the next section.

Although the training process is similar with that of machine learning models, the data-driven crop model takes a fundamentally and philosophically different learning approach from conventional neural networks. Neural networks use a generic modeling structure with a large number of parameters and rely almost exclusively on data to learn the input-output relationship without preset underlying assumptions. This approach has the potential to capture extremely subtle and insightful knowledge beyond the comprehension of human intelligence. Along with this potential benefit come two disadvantages. The first is the risk of data deficiency, either quantitatively or qualitatively, which could mislead the model into collecting biased or false knowledge and offsetting the potential benefit. The second disadvantage is the large number of parameters, which are necessary to achieve a universal approximation capability, but they make the model not only prone to overfitting but also hard to explain.

On the other hand, the structure of the data-driven crop model is determined according to human knowledge of plant physiology, which is advanced enough to qualitatively describe the crop growth process; historical data were used only to calibrate a small number of biologically meaningful parameters. For example, the fact that radiation contributes to photosynthesis is incorporated in the structure of the model, whereas historical data were used to quantitatively determine the exact rate of radiation contribution to photosynthetic yield. These genotypic parameters are independent of environmental influences, thus can be used to identify genetic characteristics of unique genotype.

### Statistics and reproducibility

The corn yield data from 1981 to 2020 in the Corn Belt area downloaded from USDA-NASS contains many missing values in different states. 47,710 county-year combinations of yield data remain after we excluded the missing values. We also utilized the means of non-missing data to impute the missing value in plant population density data.

### Reporting summary

Further information on research design is available in the Nature Portfolio Reporting Summary linked to this article.

## Supplementary information

Supplementary Information

Description of Additional Supplementary Files

Supplementary Data 1

Reporting Summary

## Data Availability

All data used in this manuscript were openly available in public domain, and the sources of these data can be found in the Method Section. Supplementary Data 1 contains the source data behind the Figs. 2, 3, 4 and 6 in the paper.

## Code availability

Code for the data-driven crop model built in this paper is available at 10.5281/zenodo.7792271.
