# Supplementary material for: A data-driven crop model for maize yield prediction
Source: Commun Biol. 2023 Apr 21;6:439. doi: 10.1038/s42003-023-04833-y (PMC10121691; doi:10.1038/s42003-023-04833-y)
Supplement: Supplementary file 1 — Supplementary Information [file 42003_2023_4833_MOESM1_ESM.pdf]

A Data-Driven Crop Model for Maize Yield Prediction

Supplementary Note 1: Input Data

Yield and Geographic Data:

- $\mathcal{TC}$: set of county-year (t, c) combinations, for which observed yield data exist
- $y_{t,c}$: observed corn yield in year t in county c.

Weather Data: The dataset included seven variables, and six (excluding snow water equivalent) are used in the model.

- $W^{\text{dayl}}_{d,t,c}$: duration of the daylight period, on day d of year t in county c, in seconds.
- $W^{\text{prcp}}_{d,t,c}$: daily total precipitation, sum of all forms converted to equivalent water, on day d of year t in county c, in mm.
- $W^{\text{srad}}_{d,t,c}$: incident shortwave radiation flux density, taken as an average over the daylight period, on day d of year t in county c, in watts per square meter.
- $W^{\text{tmax}}_{d,t,c}$: daily maximum 2-meter air temperature, on day d of year t in county c, in degrees Celsius.
- $W^{\text{tmin}}_{d,t,c}$: daily minimum 2-meter air temperature, on day d of year t in county c, in degrees Celsius.
- $W^{\text{vp}}_{d,t,c}$: water vapor pressure, on day d of year t in county c, in Pascal.

Management Data:

- $M^{\text{plant}}_{t,c}$: earliest planting day of year t in county c.
- $M^{\text{harvest}}_{t,c}$: latest harvesting day of year t in county c.
- $M^{\text{density}}_{t,c}$: plant density for corn grown in year t in county c, in number of plants per acre.
- $M^{\text{area}}_{t,c}$: planted areas of year t in county c, in acres.

Soil data: We used 10 soil variables measured in the Value Added Look Up Table (Valu1) in the database, the names and descriptions of which from [1] are summarized as follows.

- S^{aws}_{c}: available water storage in county c, expressed in mm, the volume of plant available water that the soil can store in this layer based on all map

unit components. This variable was measured by the average value of zone 5 (0-150cm).

- S_c^{tka}: thickness of soil components in county c, expressed in cm for the available water storage calculation. This variable was measured by the average value of zone 5 (0-150cm).
- S_c^{soc}: soil organic carbon stock estimate in county c, expressed in grams C per square meter to a depth of 5 cm. This variable was measured by the average value of zone 5 (0-150cm).
- S_c^{tks}: thickness of soil components in county c, expressed in cm for the soil organic carbon calculation. This variable was measured by the average value of zone 5 (0-150cm).
- $S_c^{\text{nccpi3corn}}$: national commodity crop productivity index for corn (weighted average) in county c. The values range from 0.01 (low productivity) to 0.99 (high productivity).
- $S_c^{\text{pctearthmc}}$: national commodity crop productivity index for major earthy components in county c, which are those soil series or higher level taxa components that can support crop growth.
- $S_c^{\text{rootznemc}}$: root zone depth in county c, expressed in mm, is the depth within the soil profile that commodity crop roots can effectively extract water and nutrients for growth.
- $S_c^{\text{rootznaws}}$: root zone available water storage estimate in county c, expressed in mm, is the volume of plant available water that the soil can store within the root zone based on all map unit earthy major components.
- S_c^{droughty}: drought vulnerable landscapes in county c, which comprise those map units that available water storage within the root zone for commodity crops is less than or equal to 6 inches (152 mm), expressed as “1” for a drought vulnerable soil landscape map unit or “0” for a non-droughty soil landscape map unit.
- $S_c^{\text{pwsl1pomu}}$: potential wetland soil landscapes (PWSL)in county c, expressed as the percentage of the map unit that meets the PWSL criteria.

Supplementary Note 2: Variables

Genotypic Parameters: a separate seed profile is extracted to represent the average characteristics of the genotypes grown in each county in each year. Although many genotypes are used in reality across different counties and regions with inevitable overlap, the genotypic parameter describes an average profile of a genetic portfolio that is allowed to vary by county and evolve over time. Maize growth has two phases, vegetative and reproductive. Vegetative phase subdivisions are characterized by three developmental timing epochs: planting, emergence, and peak of LAI. Subdivisions of reproductive stage can be defined as: pollination, milk, dent, and maturity. Thus, we identified 7 developmental timing epochs and 6 subdivisions in total during the maize growth process. We use $i \in \{v1, v2, v3, r1, r2, r3\}$ to represent the 6 subdivision periods between the 7 developmental timing epochs of vegetative and

reproductive stages. Genotypic parameters consist of eleven parameters under 6 growth subdivisions: (1) required growing degree days of finishing the growth stage, (2) proportion of biomass allocated to leaf, (3) proportion of biomass allocated to root, (4) proportion of biomass allocated to grain, (5) radiation use efficiency per leaf weight, (6) leaf size, (7) leaf senescence rate, (8) root water uptake rate, (9) transpiration efficiency coefficient (10) leaf cover rate per leaf weight to prevent water evaporation from soil, and (11) fraction of grain loss if waterlogging happens. And three other parameters consist of the year-county combination: (1) reference plant density, (2) base temperature for the GDD calculation, and (3) high cutoff temperature for GDD calculation.

- $g_{t,c,i}^{\text{water uptake rate}}$: maximal amount of water uptake per root weight per day of year t in county c during i subdivision period, in mm.
- $g_{t,c,i}^{\text{rue}}$: radiation use efficiency of year t in county c during subdivision period i, in joule per square meter.
- $g_{t,c,i}^{\text{trans effi}}$: transpiration efficiency coefficient of year t in county c during i subdivision period, in joules per square meter.
- $g_{t,c,i}^{\text{leaf index rate}}$: LAI rate per weight of leaves of year t in county c during subdivision period i .
- $g_{t,c,i}^{\text{leaf:biomass}}$: proportion of biomass accumulation allocated to leaf growth of year t in county c during i subdivision period, in percentage.
- $g_{t,c,i}^{\text{root:biomass}}$: proportion of biomass accumulation allocated to root growth in year t in county c during subdivision period i, in percentage.
- $g_{t,c,i}^{\text{grain:biomass}}$: proportion of biomass accumulation allocated to grain growth of year t in county c during the subdivision period i, in percentage.
- $g_{t,c,i}^{\text{leaf cover}}$: leaf cover rate per leaf weight to prevent water evaporate of year t in county c during subdivision period i, in percentage.
- $g_{t,c,i}^{\text{leaf senescence}}$: leaf senescence rate of year t in county c during subdivision period i, in percentage.
- $g_{t,c,i}^{\text{flood loss}}$: fraction of the terminal yield losses caused by the waterlogged of year t in county c during subdivision period i, in percentage.
- $g_{t,c,i}^{\text{GDD}}$: amount of growing degree days required to complete of year t in county c during i subdivision period, in Celsius degree days.
- $g_{t,c}^{\text{density}}$: reference plant density of year t in county c , in number of plants per acre.
- $g_{t,c}^{\text{tbase}}$: base temperature for GDD calculation of year t in county c , in Celsius degrees.
- $g_{t,c}^{\text{tcutoff}}$: high cutoff temperature for GDD calculation of year t in county c , in Celsius degrees.

Soil Variables: underground soil has a water holding capacity, and excess water beyond this capacity will drain at a certain rate. According to Obertson and Fukai [2], the data-driven crop model simulates a one-layer soil to track maize growth and yield. Soil water evaporates at a certain rate on a daily basis. A soil profile consists of seven parameters that define the aforementioned properties: (1) initial water level ratio, (2) soil waterlogging level, (3) soil

water holding capacity, (4) drainage rate at which excess water drains level, (5) precipitation level when water runoff occurs, (6) water runoff rate, and (7) soil water evaporation rate.

- $s_{t,c}^{\text{initial water ratio}}$: initial water ratio of the soil in county c and year t, affected by rainfall after the harvest day of last year, in percentage.
- $s_{c}^{\text{waterlogged}}$: soil waterlogged level in county c, in mm.
- $s_{c}^{\text{water capacity}}$: underground soil water holding capacity in county c, in mm.
- $s_{c}^{\text{drain rate}}$: rate at which excess water above the soil water holding capacity is drawn each day in the county c, in percentage.
- $s_{c}^{\text{runoff cutoff}}$: cutoff precipitation for runoff in county c, in mm.
- $s_{c}^{\text{runoff rate}}$: rate at which excess cutoff precipitation runoff each day in county c, in percentage.
- $s_{c}^{\text{evaporate rate}}$: rate at which soil water evaporates each day in county c, in percentage.

Intermediate Progress Variables:

- $p_{d,t,c}^{\text{tmean}}$: mean temperature on day d of year t in county c, in Celsius degrees.
- $p_{d,t,c}^{\text{GDD}}$: contribution to growing degree days from day d of year t in county c, in Celsius degrees.
- $p_{t,c,i}^{\text{GDD sum}}$: accumulated growing degree days of year t in county c in subdivision period i, in Celsius degrees.
- $p_{d,t,c}^{\text{drain}}$: amount of excess soil water that drains to deeper ground and will not be available for crop water uptake, on day d of year t in county c, in mm.
- $p_{d,t,c}^{\text{evaporate}}$: amount of soil water evaporation, on day d of year t in county c, in mm.
- $p_{d,t,c}^{\text{potential soil water}}$: potential amount of water in the soil on day d of year t in county c, in mm.
- $p_{d,t,c}^{\text{soil water}}$: amount of water in the soil on day d of year t in county c, in mm.
- $p_{d,t,c}^{\text{water supply}}$: amount of water can be uptake from soil, on day d of year t in county c, in mm.
- $p_{d,t,c}^{\text{water demand}}$: amount of water demand for normal transpiration crop process, on day d of year t in county c, in mm.
- $p_{d,t,c}^{\text{water deficit}}$: ratio of water supply to water demand, on day d of year t in county c.
- $p_{d,t,c}^{\text{vp air}}$: actual vapor pressure of air, on day d of year t in county c, in KPa.
- $p_{d,t,c}^{\text{vp sat}}$: saturated vapor pressure, on day d of year t in county c, in KPa.
- $p_{d,t,c}^{\text{vpd}}$: vapor pressure deficit, on day d of year t in county c.
- $p_{d,t,c}^{\text{MJ}}$: daily total radiation, on day d of year t in county c, in MJ.
- $p_{d,t,c}^{\text{LAI}}$: LAI, on day d of year t in county c.
- $p_{t,c,i}^{\text{waterlogged}}$: number of days that the soil water level excesses the waterlogged level during i subdivision period of year t in county c.
- $p_{d,t,c}^{\text{dbiomass}}$: amount of daily biomass accumulation, on day d of year t in county c, in mg/ha.

- $p^{\text{leaves}}_{d,t,c}$: weight of leaves, on day d of year t in county c, in mg/ha.
- $p^{\text{roots}}_{d,t,c}$: weight of roots, on day d of year t in county c, in mg/ha.
- $p^{\text{grain}}_{d,t,c}$: weight of grain, on day d of year t in county c, in mg/ha.

Supplementary Note 3: Crop Model Function

Step 1: Initialization for $d = 0$ for all (t, c).

$$p^{\text{soil water}}_{0,t,c} = s^{\text{aws}}_{c} \cdot s^{\text{initial water ratio}}_{t,c} \tag{C.1}$$

$$p_{0,t,c} = 0,\ \forall \text{ other intermediate progress variables} \tag{C.2}$$

Step 2: Simulation for days before planting, when $1 \le d < M^{\textbf{plant}}_{t,c}$, for all (t, c).

$$p^{\text{tmean}}_{d,t,c} = \frac{W^{\text{tmax}}_{d,t,c} + W^{\text{tmin}}_{d,t,c}}{2} \tag{C.3}$$

$$p^{\text{MJ}}_{d,t,c} = W^{\text{dayl}}_{d,t,c} \cdot W^{\text{srad}}_{d,t,c}/1000000 \tag{C.4}$$

$$p^{\text{vp air}}_{d,t,c} = W^{\text{vp}}_{d,t,c}/1000 \tag{C.5}$$

$$p^{\text{vp sat}}_{d,t,c} = 610.7 \cdot 10^{(7.5 \cdot p^{\text{tmean}}_{d,t,c}/(237.3 + p^{\text{tmean}}_{d,t,c}))}/1000 \tag{C.6}$$

$$p^{\text{vpd}}_{d,t,c} = p^{\text{vp\_air}}_{d,t,c} - p^{\text{vp\_sat}}_{d,t,c} \tag{C.7}$$

$$p^{\text{evaporate}}_{d,t,c} = p^{\text{MJ}}_{d,t,c} \cdot s^{\text{evaporate rate}}_{c} \tag{C.8}$$

$$p^{\text{runoff water}}_{d,t,c} = \max\{p^{\text{prcp}}_{d,t,c} - s^{\text{runoff cutoff}}_{c}, 0\} \cdot s^{\text{runoff rate}}_{c} \tag{C.9}$$

$$p^{\text{potential soil water}}_{d,t,c} = p^{\text{soil water}}_{d-1,t,c} + W^{\text{prcp}}_{d,t,c} - p^{\text{runoff water}}_{d,t,c} - p^{\text{evaporate}}_{d,t,c} \tag{C.10}$$

$$p^{\text{drain}}_{d,t,c} = \max\{p^{\text{potential soil water}}_{d,t,c} - s^{\text{water capacity}}_{c}, 0\} \tag{C.11}$$

$$p^{\text{soil water}}_{d,t,c} = p^{\text{potential soil water}}_{d,t,c} - p^{\text{drain}}_{d,t,c} \tag{C.12}$$

Step 3: Simulation for days during vegetative stages, when $M^{\textbf{plant}}_{t,c} \le d \le M^{\textbf{harvest}}_{t,c}$ and $p^{\textbf{GDD sum}}_{t,c,i} \le g^{\textbf{GDD}}_{t,c,i}$, for all (t, c) and $i \in \{v1, v2, v3\}$. Note, here we initialize $p^{\textbf{roots}}_{d,t,c}$ and $p^{\textbf{leaves}}_{d,t,c}$ as two positive values to represent the emergence status

Equations (C.3)-(C.7)

$$p^{\text{GDD}}_{d,t,c} = \frac{\min\{W^{\text{tmax}}_{d,t,c}, g^{\text{tcutoff}}_{t,c}\} + \max\{W^{\text{tmin}}_{d,t,c}, g^{\text{tbase}}_{t,c}\}}{2} - g^{\text{tbase}}_{t,c} \tag{C.13}$$

$$p^{\text{GDD sum}}_{t,c,i} = p^{\text{GDD sum}}_{t,c,i} + p^{\text{GDD}}_{d,t,c} \tag{C.14}$$

$$p^{\text{LAI}}_{d,t,c} = p^{\text{leaves}}_{d-1,t,c} \cdot g^{\text{leaf index rate}}_{t,c,i} \cdot \frac{M^{\text{density}}_{t,c}}{g^{\text{density}}_{t,c}} \tag{C.15}$$

$$p^{\text{potential biomass}}_{d,t,c} = g^{\text{rue}}_{t,c,i} \cdot p^{\text{LAI}}_{d,t,c} \tag{C.16}$$

$$p^{\text{water supply}}_{d,t,c} = \max\{g^{\text{water uptake rate}}_{t,c,i} \cdot p^{\text{soil water}}_{d,t,c} \cdot p^{\text{roots}}_{d,t,c}, 0\} \tag{C.17}$$

$$p^{\text{water demand}}_{d,t,c} = p^{\text{potential biomass}}_{d,t,c} / p^{\text{vpd}}_{d,t,c} / g^{\text{trans effi}}_{t,c,i} \cdot \frac{M^{\text{density}}_{t,c}}{g^{\text{density}}_{t,c}} \tag{C.18}$$

$$p^{\text{water deficit}}_{d,t,c} = \min\{p^{\text{water supply}}_{d,t,c} / p^{\text{water demand}}_{d,t,c}, 1\} \tag{C.19}$$

$$p^{\text{dbiomass}}_{d,t,c} = p^{\text{potential biomass}}_{d,t,c} \cdot p^{\text{water deficit}}_{d,t,c} \tag{C.20}$$

$$p^{\text{evaporate}}_{d,t,c} = p^{\text{MJ}}_{d,t,c} \cdot s^{\text{evaporate rate}}_{c} \cdot (1 - g^{\text{leaf cover}}_{t,c,i} \cdot p^{\text{leaves}}_{d,t,c}) \tag{C.21}$$

$$p^{\text{runoff water}}_{d,t,c} = \max\{p^{\text{prcp}}_{d,t,c} - s^{\text{runoff cutoff}}_{c}, 0\} \cdot s^{\text{runoff rate}}_{c} \tag{C.22}$$

$$\begin{aligned} p^{\text{potential soil water}}_{d,t,c} &= p^{\text{soil water}}_{d-1,t,c} + W^{\text{prcp}}_{d,t,c} - p^{\text{runoff water}}_{d,t,c} - p^{\text{evaporate}}_{d,t,c} \\ &\quad - p^{\text{water demand}}_{d,t,c} \cdot p^{\text{water deficit}}_{d,t,c} \end{aligned} \tag{C.23}$$

$$p^{\text{drain}}_{d,t,c} = \max\{p^{\text{potential soil water}}_{d,t,c} - s^{\text{water capacity}}_{c}, 0\} \tag{C.24}$$

$$p^{\text{soil water}}_{d,t,c} = p^{\text{potential soil water}}_{d,t,c} - p^{\text{drain}}_{d,t,c} \tag{C.25}$$

$$\begin{aligned} p^{\text{waterlogged}}_{t,c,i} &= p^{\text{waterlogged}}_{t,c,i} + \\ &\quad \min\{\max\{p^{\text{potential soil water}}_{d,t,c} - s^{\text{waterlogged}}_{c}, 0\}, 1\} \end{aligned} \tag{C.26}$$

$$p^{\text{roots}}_{d,t,c} = p^{\text{roots}}_{d-1,t,c} + p^{\text{dbiomass}}_{d,t,c} \cdot g^{\text{root:biomass}}_{t,c,i} \tag{C.27}$$

$$p^{\text{leaves}}_{d,t,c} = p^{\text{leaves}}_{d-1,t,c} + p^{\text{dbiomass}}_{d,t,c} \cdot g^{\text{leaf:biomass}}_{t,c,i} \tag{C.28}$$

Step 4: Simulation for days during reproductive stages, when $M^{\textbf{plant}}_{t,c} \le d \le M^{\textbf{harvest}}_{t,c}$ and $p^{\textbf{GDD sum}}_{t,c,i} \le g^{\textbf{GDD}}_{t,c,i}$, for all (t,c) and $i \in \{r1, r2, r3\}$.

Equations (C.3)-(C.7), (C.13)-(C.26)

$$p^{\text{grain}}_{d,t,c} = p^{\text{grain}}_{d-1,t,c} + p^{\text{biomass}}_{d,t,c} \cdot g^{\text{grain:biomass}}_{t,c,i} \tag{C.29}$$

$$p^{\text{leaves}}_{d,t,c} = p^{\text{leaves}}_{d-1,t,c} \cdot (1 - g^{\text{senescence}}_{t,c,i}) \tag{C.30}$$

Step 5:

$$\hat{y}_{t,c} = p^{\text{grain}}_{M^{\text{harvest}},t,c} \tag{C.31}$$

According to the descriptive modeling framework stated in the Method Section, the phenology clock for maize are described in Equations (C.13) and (C.14). Equations (C.8) - (C.12) and (C.21) - (C.25) represent the soil water module with and without the consideration of crop water intake, respectively. The potential water uptake by maize is described in Equation (C.18). The radiation and daily biomass production modules can be explained by Equations

(C.15) and (C.16). The metabolic process and organ growth modules are summarized in Equations (C.17), (C.27) - (C.30). Equations (C.19) and (C.26) state the drought and flooding stress, respectively. Below shows more detailed explanation of the crop model function.

In step 1, Equation (C.1) defines the initial soil water level at the beginning of the year. Equation (C.2) initializes all the other intermediate progress variables as 0. Equations (C.3) - (C.12) simulate the intermediate variables before the planting date. Equation (C.3) calculates the mean temperature with the respective maximum and minimum temperatures. Equations (C.4) and (C.5) calculate the daily total radiation and air vapor pressure according to the descriptions on the Daymet website [3]. Equation (C.6) calculates the saturated vapor pressure with the instruction [4]. Equation (C.7) gives us the vapor pressure deficit that can be used to calculate the water demand of maize plant in the next step. Equations (C.8) - (C.12) update the soil water level with a comprehensive combination of evaporation, runoff and drain water.

Equations (C.13) and (C.14) describe how the growing degree days are accumulated when considering the likelihood that a high temperature cutoff is occurring. Equation (C.15) shows how to generate a LAI value with a given leaf weight. Equation (C.16) calculates the maximum potential biomass that can be generated under ideal water supply conditions. Equations (C.17) - (C.19) indicate how water deficit index is computed. The water supply is determined by the soil water level and roots. Water demand is calculated as defined by the APSIM [5]. Using water deficit index, we can obtain the actual daily biomass generated by the photosynthesis process in Equation (C.20). Equations (C.21) - (C.25) are similar to Equations (C.8) - (C.12) and used to calculate the soil water level during the maize growth. Equation (C.26) records the number of days that waterlogging occurs. Equations (C.27) and (C.28) describe how root and leaf growth with the partial of produced biomass. In the reproductive stage, the root is no longer growing, the leaf senescence is shown in equation (C.30), and the grain begins to accumulate biomass as described in equation (C.29).

The data-driven crop model is a complex nonlinear optimization problem that is not readily solvable by standard machine learning algorithms. Herein, we present a heuristic algorithm that can efficiently extract a high quality solution (without guarantee of global optimality) for the soil profile (s, L) and the genetics profile (g) parameters for all year-county combinations. The strategy is to iteratively update one of these profile variables at a time while keeping the other fixed, which is done by exploring a small neighborhood with different step sizes. As such, model (8)-(11) was solved as a simulation optimization problem. Detailed steps of the heuristic algorithm are explained as follows.

Step 0: Initialization. initial incumbent solutions (s^*, L^*) and $g^*_{t,c}$ are estimated and used for all (t, c). Go to step 1.

Step 1: Update (s^*, L^*). randomly select a variable in (s^*, L^*) and try increasing and decreasing its value with different step sizes. Evaluate the objective (8) for all new values of this variable. Update the incumbent solution (s^*, L^*) with the new value that resulted in the lowest RMSE. Go to step 2.

Step 2: Update $g^*_{t,c}$. for all (t, c), randomly select a variable in $g^*_{t,c}$ and try increasing and decreasing its value with different step sizes. Evaluate the objective (8) for all new values of this variable. Update the incumbent solution $g^*_{t,c}$ with the new value that resulted in the lowest RMSE.

Terminate the algorithm if neither (s^*, L^*) nor $g^*_{t,c}$ for any (t, c) has been updated or the running time limit has been reached; otherwise go back to Step 1 for a new iteration.

References

[1] USDA: The Gridded Soil Survey Geographic (2020). https://www.nrcs.usda.gov/wps/portal/nrcs/site/soils/home Accessed 2022-05-03

[2] Robertson, M., Fukai, S.: Comparison of water extraction models for grain sorghum under continuous soil drying. Field Crops Research **36**(2), 145–160 (1994)

[3] Thornton, P.E., Thornton, M.M., Mayer, B.W., Wei, Y., Devarakonda, R., Vose, R.S., Cook., R.B.: Daymet: Daily Surface Weather Data on a 1-km Grid for North America, Version 3. (2020). https://doi.org/10.3334/ORNLDAAC/1328 Accessed 2022-05-03

[4] Metsaots, R.: National Agricultural Statistical Service (2018). https://betterorganix.com/blog/what-is-how-to-calculate-vapour-pressure-deficit/ Accessed 2022-05-03

[5] Balboa, G.R., Archontoulis, S., Salvagiotti, F., Garcia, F.O., Stewart, W., Francisco, E., Prasad, P.V., Ciampitti, I.A.: A systems-level yield gap assessment of maize-soybean rotation under high-and low-management inputs in the western us corn belt using apsim. Agricultural Systems **174**, 145–154 (2019)
