# Supplementary material for: A data-driven crop model for maize yield prediction
Source: Commun Biol. 2023 Apr 21;6:439. doi: 10.1038/s42003-023-04833-y (PMC10121691; doi:10.1038/s42003-023-04833-y)
Supplement: Supplementary file 2 — Description of Additional Supplementary Files [file 42003_2023_4833_MOESM2_ESM.pdf]

Description of Additional Supplementary Files

File name: Supplementary Data 1

Description: The source data behind the figures (2, 3, 4, 6) in the paper
